# Design and development of spectrophotometric enzymatic cyanide assays

**DOI:** 10.1007/s00216-024-05703-0

**Published:** 2024-12-20

**Authors:** Katarína Šťastná, Ludmila Martínková, Lenka Rucká, Barbora Křístková, Romana Příhodová, Pavla Bojarová, Miroslav Pátek

**Affiliations:** 1 Institute of Microbiology of the Czech Academy of Sciences, CZ-142 00 Prague, Czech Republic; 2 Department of Biochemistry, Faculty of Sciences, Charles University, CZ-128 44 Prague, Czech Republic; 3 Faculty of Food and Biochemical Technology, University of Chemistry and Technology, Prague, CZ-166 28 Prague, Czech Republic; 4 Department of Health Care Disciplines and Population Protection, Faculty of Biomedical Engineering, Czech Technical University in Prague, nám. Sítná 3105, CZ-272 01 Kladno, Czech Republic

**Keywords:** Free cyanide, Cyanide dihydratase, Cyanide hydratase, Formamidase, Formate dehydrogenase, Enzymatic assays

## Abstract

Determination of free cyanide (fCN) is required for various industrial, environmental, food, and clinical samples. Enzymatic methods are not widely used in this field despite their selectivity and mild conditions. Therefore, we present here a proof of concept for new spectrophotometric enzymatic assays of fCN. These are based on the hydrolysis of fCN affording the readily detectable NADH. fCN is hydrolyzed either in one step by cyanide dihydratase (CynD) or in two steps by cyanide hydratase (CynH) and formamidase (AmiF). An advantage of the latter route is the higher activity of CynH and AmiF compared to CynD. In both cases, the resulting formate is then transformed by an NAD-dependent formate dehydrogenase (FDH). The NADH thus formed is quantified colorimetrically using a known method based on a reduction of a tetrazolium salt (WST-8) with NADH. The developed assays of fCN are selective except for formic acid interference, proceed under mild conditions, and, moreover, fCN is detoxified during the reactions. The assays proceeded in a microtiter plate format. The limit of detection (LOD) and the limit of quantification (LOQ) were lower for the three-enzyme (CynH-AmiF-FDH) method (7.00 and 21.2 µmol/L, respectively) than for the two-enzyme (CynD-FDH) method (10.7 and 32.4 µmol/L, respectively). In conclusion, the new fCN assays presented in this work are selective, high-throughput, do not require harsh conditions, and use only small amounts of chemicals and enzymes.

**Graphical Abstract:**

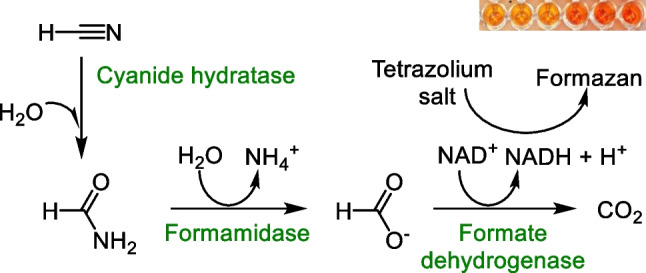

**Supplementary Information:**

The online version contains supplementary material available at 10.1007/s00216-024-05703-0.

## Introduction

Free cyanide (fCN) is the sum of cyanide ions (CN^−^) and hydrogen cyanide (HCN). The latter, which tends to form highly toxic vapors, prevails in fCN solutions especially at pH ≤ 8.5 [1]. fCN is generally more toxic than metal-complexed cyanides which, however, can liberate fCN under certain conditions [1]. fCN is one of highly threatening environmental contaminants and originates from several industries (gold and silver mining, petrochemistry, coal coking, fine chemical production, work-up of cyanogenic food plants, etc.) but also other resources such as motor vehicle exhaust gases and smoking. For example, extraction of gold and silver ores requires bulk amounts of simple cyanide (NaCN) and leaves significant fCN residues in the wastes (“tailings”) [2–4].

Determination of fCN is required for various types of samples—industrial wastewaters, natural water, drinking water, cyanogenic plants and foods produced from them, body fluids and tissues, etc. Routine fCN tests are primarily based on colorimetric reactions. A well-known example is the reaction of HCN with picric acid (2,4,6-trinitrophenol) [5–7]. The commercial Spectroquant® Cyanide Reagent Test is based on chlorinating cyanide ions to cyanogen chloride (CNCl) followed by the reaction of CNCl and 1,3-dimethylbarbituric acid [8]. Recently, new methods were developed using a cobalamin (vitamin B_12_) derivative [9] (a fluorimetric method) or Ag and Ag-Au nanoparticles (colorimetric methods) [10, 11]. Other methods include a chromatographic separation of CN^−^ followed by pulsed amperometric detection [12], GC–MS [13], or ion-selective electrodes [14, 15].

Cyanide-converting enzymes have been proposed for use in fCN biosensors and biotests [16]. For example, the transformation of fCN was catalyzed by cyanide dihydratase (CynD), which is a type of nitrilase (EC 3.5.5.1) and hydrolyzes HCN to ammonia and formate. fCN was quantified on the basis of ammonia production [17, 18] or NADH production by formate dehydrogenase (FDH, EC 1.17.1.9) [19]. NADH was determined amperometrically [19], spectrophotometrically at 340 nm [16] or utilized in a coupled reaction catalyzed by salicylate hydroxylase (SHL, EC 1.14.13.1.). SHL recycled NADH along with oxygen consumption. The concomitant decrease in current was measured using the Clark electrode (Pt working electrode, Ag/AgCl reference electrode) [16].

Enzymatic assays based on a spectrophotometric measurement of NADH are selective and sensitive. Commercial assay kits based on NADH quantitation at 340 nm have been developed, e.g., for L- or D-lactic acid or formic acid [20, 21]. In addition, the sensitivity of NAD(P)H detection and quantitation can be significantly enhanced by the addition of an electron mediator and a water-soluble tetrazolium (WST) salt that is reduced by NAD(P)H to formazan [22, 23]. Assays of this type have been developed to determine the activity of dehydrogenases [23]. Here, we investigated whether this type of assay could be used for fCN determination.

**Fig. 1:**
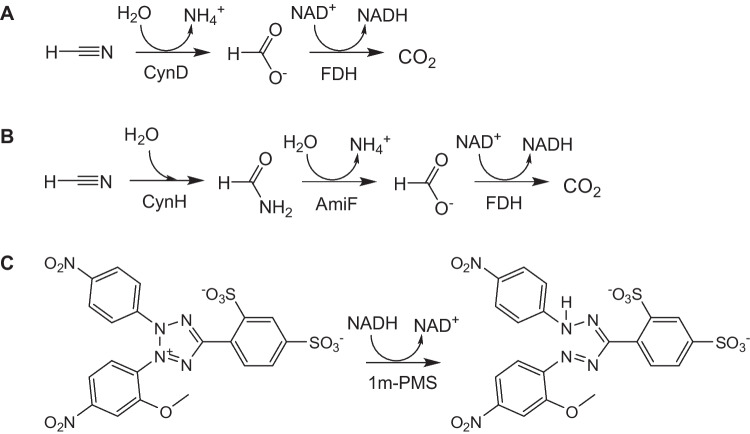
Proposed cyanide assays based on multistep reactions: **A** a cyanide dihydratase (CynD)–formate dehydrogenase (FDH) catalyzed reaction or **B** a cyanide hydratase (CynH)–formamidase (AmiF)–FDH catalyzed reaction. **C** Detection of NADH by a previously described colorimetric method [22, 23]. The water-soluble tetrazolium salt WST-8 is reduced, in the presence of 1-methoxy-5-methylphenazinium methyl sulfate (1-mPMS), to color formazan, which is detected at 460 nm

In this work, we have designed and developed new colorimetric enzymatic assays of fCN. These assays are based on a multistep reaction in which fCN is hydrolyzed to formic acid and NADH is formed in a coupled reaction. The reactions can be catalyzed by either CynD and FDH [16–19] (Fig. 1A) or by cyanide hydratase (CynH; EC 4.2.1.66), formamidase (AmiF; EC 3.5.1.49), and FDH [16] (Fig. 1B). In both cases, NADH is determined with a WST salt, 2-(2-methoxy-4-nitrophenyl)−3-(4-nitrophenyl)−5-(2,4-disulfophenyl)−2*H* tetrazolium monosodium salt (WST-8), as published previously [22, 23] (Fig. 1C).

The reaction that uses CynH as the first enzyme (Fig. 1B) will require one more enzyme (AmiF) in comparison with the reaction that starts with CynD (Fig. 1A). Nevertheless, a potential advantage of the former reaction is higher activities of CynH and AmiF [24] compared to CynD [25]. The reactions should be carried out at a sufficiently high pH to avoid losses of fCN. In this respect, both CynD and CynH are acceptable, as at least some of them maintain sufficient activity up to high pH values such as pH ≈ 9.5. The combination of purified CynH and AmiF from different sources was already shown to be feasible in our recent work that focused on the bioremediation of fCN [24].

## Materials and methods

### Chemicals

The standards and reaction mixture ingredients were purchased from standard suppliers: formamide (99.5%) for analysis (Acros Organics (Thermo Scientific), USA), KCN (≥ 96%)-ACS reagent (Sigma-Aldrich, USA); nicotinamide adenine dinucleotide (NAD) hydrate for biochemistry (Sigma-Aldrich, USA); NADH disodium salt trihydrate (Abcam, USA); WST-8 (MedChemExpress, USA); 1-methoxy-5-methylphenazinium methyl sulfate (1-mPMS; Cayman Chemical, USA).

### Enzymes

CynD was the previously reported artificial variant CynD_pum-stut_ [25]. The enzyme contained an *N*-terminal His_6_-tag and was produced in *E. coli* BL21 (DE3). The previously described production of this enzyme [25] was partially modified. The culture was grown in 200 mL of 2xYT medium with kanamycin (30 μg/mL) at 37 °C. Optical density (OD_600_) was monitored at 600 nm. When OD_600_ reached 1.0, isopropyl β-D-thiogalactopyranoside (IPTG) was supplemented to a final concentration of 0.02 mmol/L, and cultivation was continued at 20 °C for 18 h until OD_600_ reached approximately 10. Harvested cells were washed with sodium phosphate buffer (50 mmol/L, pH 7.0), supplemented with NaCl (300 mmol/L) and phenylmethylsulfonyl fluoride (PMSF; 0.1 mmol/L), and sonicated six times for 30 s at 4 °C in sonicator Bandelin Sonopuls HD2200 at 20% of maximum power output. After removing cell debris by centrifugation (2 × 15 min, 27 000 g, 4 °C), the cell-free extract (CFE) was purified on TALON® Metal Affinity Resin (Clontech, USA). The columns (2 × 1 mL) were washed with 20 mL of sodium phosphate buffer (20 mmol, pH 7.5) supplemented with NaCl (100 mmol/L) and PMSF (0.1 mmol/L). CynD was eluted with the same buffer containing imidazole (50–200 mmol/L). Selected fractions were pooled, and buffer was exchanged for sodium phosphate buffer (20 mmol/L, pH 7.6) using Amicon Ultra-30 K filter units. The purity of the enzyme was monitored using SDS-PAGE with a 12% polyacrylamide gel (see Electronic Supplementary Material, Fig. S1).

CynH (GenBank: KZV9269) from *Exidia glandulosa* was produced in *Escherichia coli* Origami B(DE3) as a fusion protein with a C-terminal His_6_-tag. Cultivation and purification were carried out, and the activity was determined as described previously [26]. Briefly, cultivation proceeded in 2 × YT medium with ampicillin (100 μg/mL) at 37 °C until OD_600_ reached 1.0. IPTG was added to a concentration of 0.02 mmol/L, and temperature was decreased to 20 °C. The cells were harvested after 20 h cultivation at 20 °C, and CFE was prepared by cell sonication. The enzyme was purified in one step using Talon® Metal Affinity Resin.

AmiF (pdb code: 5G3O_A) from *Bacillus cereus* (enzyme BceAmiF) was produced in *E. coli* BL21(DE3) as a fusion protein with a C-terminal His_6_-tag. The cultivation and purification methods were as described for CynH (see above).

CynD, CynH and AmiF were stored on ice and could be used within several months under these conditions. FDH from *Candida boidinii* was purchased from Megazyme Ltd.

### Activity assays

The activity of CynD was determined as follows: reactions were carried out using a Thermomixer Eppendorf Compact at 30 °C and 350 rpm. Reaction mixtures (0.5 mL) contained glycine/NaOH buffer (100 mmol/L, pH 9.0, pH 9.5 or pH 10.0) and 5–10 μg of the purified enzyme. The mixture was preincubated for 5 min, and the reaction was started with KCN (5 mmol/L) added from a stock solution of KCN (100 mmol/L) in glycine/NaOH buffer (100 mmol/L, pH 9.0). The mixture was incubated for further 5 min, and the reaction was stopped by adding NaOH (200 mmol/L) to a final concentration of 130 mmol/L. Residual fCN was determined using a picric acid reagent as described previously [26]. One unit of CynD activity is defined as the amount of enzyme that converts 1 μmol of fCN/min under these conditions.

The activity of CynH was determined as described previously [26]. One unit of the CynH activity is defined as the amount of enzyme that converts 1 µmol of HCN per min at pH 9.0 and 30 °C.

The activity of AmiF was determined as described previously [24], but with Tris/HCl buffer (100 mmol/L, pH 7.6). In this study, one unit of AmiF activity is defined as the amount of enzyme that converts 1 µmol of formamide per min at pH 7.6 and 30 °C.

### Enzyme reactions

#### Transformation of formamide by formamidase and formate dehydrogenase

Formamide solutions (0.01–0.20 mmol/L) were prepared in glycine/NaOH buffer (100 mmol/L, pH 9.5). The reaction mixture (0.2 mL) contained 0.1 mL of the formamide solution (final concentration 0.005–0.10 mmol/L), 0.02 mL of Tris/HCl buffer (1 mol/L, pH 7.1), purified AmiF (1 U), FDH (0.2 U), NAD^+^ (5 mmol/L), WST-8 (0.2 mmol/L), and 1-mPMS (0.008 mmol/L). The reactions were performed in a 96-well microplate using a microtitration plate reader Tecan Sunrise™ controlled by the software Magellan™ (Tecan). The reaction mixture was preincubated at 37 °C for 5 min. The reaction was started with FDH and proceeded at 37 °C for 40 min. The plates were shaken for 10 s prior to each read-out (each 10 min). The production of formazan was determined at 460 nm.

#### Transformation of cyanide by cyanide dihydratase and formate dehydrogenase

The first reaction was performed using a Thermomixer Eppendorf Compact (30 °C, 350 rpm). The reaction mixture (0.5 mL) contained glycine/NaOH buffer (100 mmol/L, pH 9.5), a KCN standard (0.0192–0.096 mmol/L), and 10 U of purified CynD. Samples were withdrawn after 20 min and directly used for the second reaction which was performed in a 96-well microplate using a microtitration plate reader (see above). The reaction mixture (0.20 mL) contained the sample (0.12 mL), 0.02 mL of Tris/HCl buffer (1 mol/L, pH 7.1), FDH (0.2 U), NAD^+^ (5 mmol/L), WST-8 (0.2 mmol/L), and 1-mPMS (0.008 mmol/L). The reaction mixture was preincubated at 37 °C for 5 min. The reaction was started with FDH and proceeded at 37 °C for 40 min. The plates were shaken for 10 s prior to each read-out (each 10 min). The production of formazan was determined at 460 nm.

#### Transformation of cyanide by cyanide hydratase, formamidase, and formate dehydrogenase

The first reaction was carried out under the same conditions but CynD was replaced by CynH (10 U). The second reaction was also performed as described above, but AmiF (1 U) was added to the reaction mixture. The absorbance at 460 nm was monitored for 70 min.

### Determination of detection and quantification limits

Calibration curves were constructed by plotting the absorbance at 460 nm against the concentration of standard (KCN or formamide). Limit of detection (LOD) and limit of quantification (LOQ) were calculated according to the following equations [27]:

$$LOD=3.3\;\sigma/S,$$

$$LOQ=10\;\sigma/S,$$

where *σ* is the standard deviation of response (STEYX function in MS Excel) and *S* is the slope of the calibration curve (SLOPE function in MS Excel).

## Results and discussion

### Enzyme selection and assay design

The first reaction in the proposed multistep reactions of fCN required a suitable cyanide-transforming enzyme. In principle, this enzyme can be a CynD or a CynH [16]. However, wild-type CynDs are almost inactive at pH 9.0 or higher. Fortunately, CynD mutants active at pH 9.0–9.5 were reported [25]. One of the best was the mutant CynD_pum-stut_, which is a hybrid of two wild-type CynDs [25]. Its activity was maximum at pH 9.0 (approximately 22 U/mg). It still maintained about one half of its maximum activity at pH 9.5, as previously determined for KCN (4 mmol/L) as substrate at room temperature [25]. In this work, CynD_pum-stut_ was produced and purified according to the previous study [25] with minor modifications (see “Materials and methods” section). CynD was purified in one step by metal affinity chromatography, and the resulting enzyme was homogeneous according to SDS-PAGE (see Electronic Supplementary Material, Fig. S1). Its activity was determined for KCN (5 mmol/L) at 30 °C. The specific activities determined under these conditions were higher than in the previous study, i.e., approximately 39.8 ± 2.0 U/mg and 15.5 ± 1.7 U/mg at pH 9.0 and pH 9.5, respectively. In both studies, pH 10.0 was found to be unsuitable for the enzyme activity.

Alternatively, CynHs can be used in the first step, while the product is formamide. CynHs are known for their excellent specific activities at alkaline pH. For example, a CynH from *Exidia glandulosa* (GenBank: KZV92691.1) exhibited a specific activity of 784 ± 32 U/mg for 25 mmol KCN/L at pH 9.0 and 30 °C, and it still retained 73% of its maximum activity at pH 10.3 [26]. However, this alternative will require to use an additional enzyme, AmiF, to convert formamide to formic acid.

The enzyme AmiF originating from *B. cereus* (pdb code: 5G3O_A) was reported previously [28] and was recently used by us to convert formamide in a two-step degradation of fCN [24]. Its maximum activity was 2800 ± 500 U/mg at pH 6.0 and 50 °C according to the previous work [28]. Recently, we investigated the activity of this enzyme at pH 8.0 − 10.0 and 30 °C as conditions that will be more appropriate for the transformation of fCN. The activity at pH 8.0 and 30 °C was 1440 ± 200 U/mg, of which ≈ 55% and ≈ 40% was maintained at pH 9.0 and 9.5, respectively [24]. Thus, CynH and AmiF are also compatible in terms of their specific activities.

The multistep reaction was proposed as follows: In the first step, fCN is converted to formic acid by the CynD at pH 9.5. The samples are then transferred to the second reaction mixture to convert formic acid with FDH. The enzyme FDH (commercial) was from *C. boidinii* and its optima were pH 7.6 and 37 °C according to the manufacturer [29]. These parameters were disadvantageous for performing the whole cascade in one pot, as these conditions are not quite compatible with handling fCN.

The other multistep reaction is started with the CynH-catalyzed reaction at pH 9.5. The next two reactions catalyzed by AmiF and FDH are performed in one pot. AmiF is principally compatible with both CynH and FDH. Here, we decided to combine it with FDH due to the similar pH optima of FDH and AmiF. In addition, fCN was previously found to decrease the activity of AmiF [24]. This was another reason for using AmiF in the second step, in which the reaction mixture presumably does not contain any significant amounts of fCN.

### Determination of free cyanide using a cyanide dihydratase–formate dehydrogenase cascade

The performance of the CynD − FDH cascade was investigated using KCN solutions of known concentrations (0.0192–0.096 mmol/L). These were first converted by the CynD, and the samples were then transferred to the second reaction mixture with FDH. Although NADH is commonly detected at 340 nm (see Electronic Supplementary Material, Fig. S2A), this method was not quite suitable for low concentrations of NADH. Therefore, the previously described colorimetric method based on WST-8 reduction by NADH [22, 23] was used (see Electronic Supplementary Material, Fig. S2B). The absorbance of formazan resulting from the reduction of WST-8 was monitored at 460 nm.

**Fig. 2:**
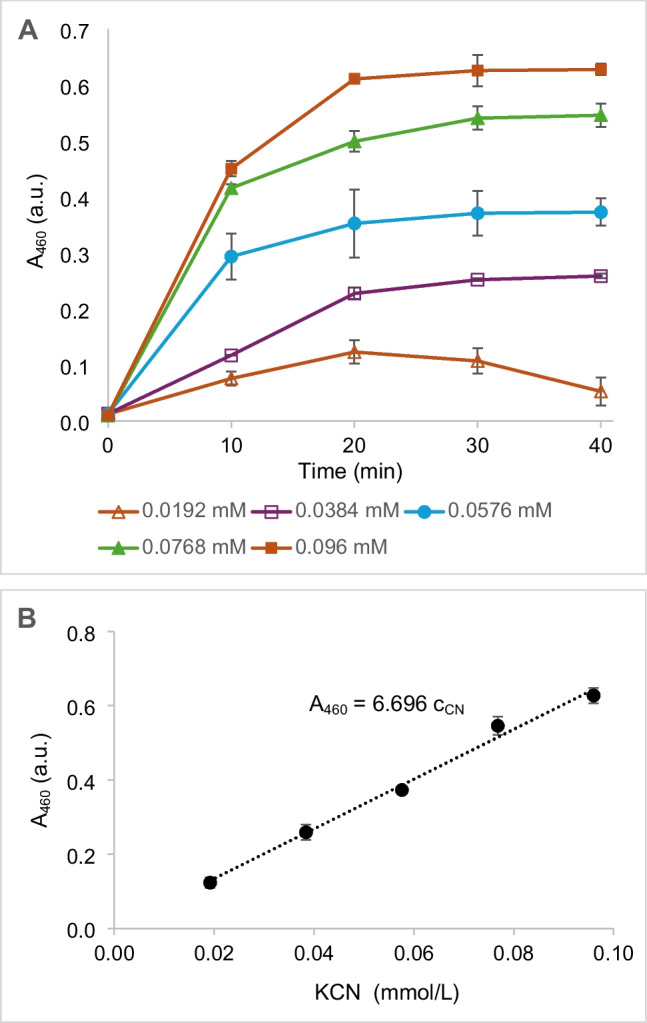
**A** Development of absorbance of formazan at 460 nm (A_460_, absorbance units, a.u.) in the reactions with various concentrations of KCN (c_CN_, mmol/L). **B** Maximum absorbance (A_460_, a.u.) reached for each concentration of KCN. The two-step reaction of KCN was catalyzed by cyanide dihydratase (CynD) and formate dehydrogenase (FDH). The first step proceeded at 30 °C for 20 min. The reaction mixture (0.5 mL) contained glycine/NaOH buffer (100 mmol/L, pH 9.5), KCN (0.0192–0.096 mmol/L) and CynD (10 U). The second step proceeded at 37 °C. The reaction mixture (0.20 mL) contained 0.12 mL of sample from the first step, 0.02 mL of Tris/HCl buffer (1 mol/L, pH 7.1), FDH (0.2 U), NAD^+^ (5 mmol/L), WST-8 (0.2 mmol/L), and 1-mPMS (0.008 mmol/L)

The absorbance (A_460_) reached its maximum after 20–40 min, depending on the concentration of standard (KCN; Fig. 2A). The maximum absorbance for each concentration was read out and plotted against the KCN concentration. The linearity of the response for ≈ 0.02 through ≈ 0.1 mmol KCN/L was satisfactory (Fig. 2B). The LOD and LOQ were calculated from the standard deviation of the response and the slope of the calibration curve (see Materials and methods). The LOD and LOQ were 10.7 μmol/L (0.28 mg CN^−^/mL) and 32.4 μmol/L (0.84 mg CN^−^/mL), respectively (Table 1).

**Table 1:** Limit of detection (LOD) and limit of quantification (LOQ) for the determination of free cyanide supplemented as KCN (Figs. 2B, 4B), and formamide (Fig. 3B)

Compound	Enzymes used	STEYX function	SLOPE function	LOD (µmol/L)	LOQ (µmol/LM)
Formamide	AmiF-FDH	0.0965	24.225	13.1	39.8
KCN	CynD-FDH	0.0217	6.696	10.7	32.4
KCN	CynH-AmiF-FDH	0.0262	12.374	7.00	21.2

### Determination of formamide using a formamidase–formate dehydrogenase cascade

**Fig. 3:**
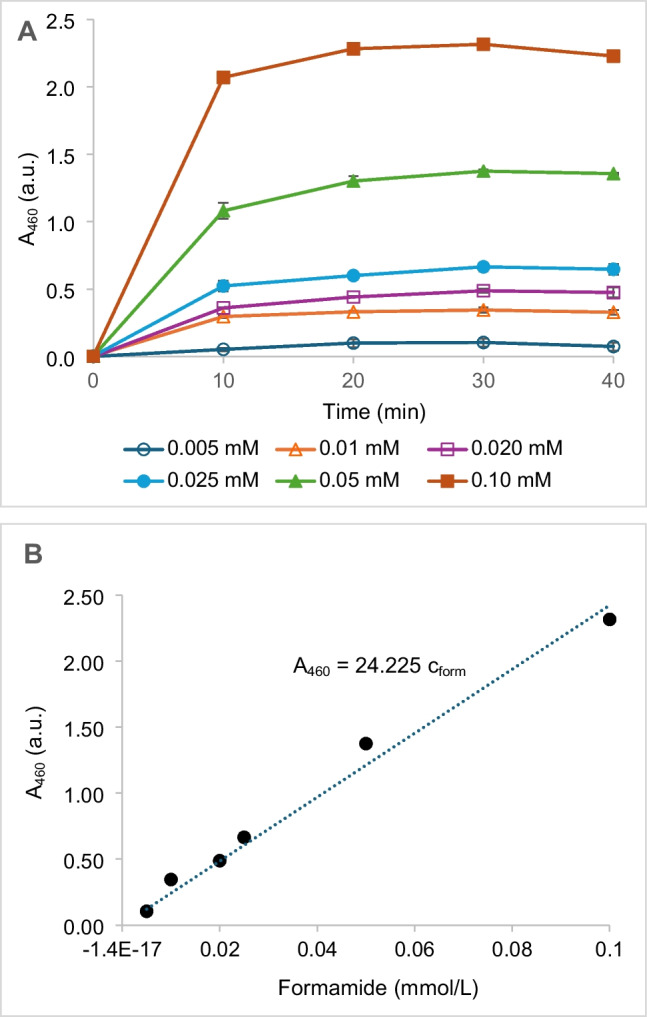
**A** Development of absorbance of formazan at 460 nm (A_460_, absorbance units, a.u.) in the reactions of various concentrations of formamide (c_form_, mmol/L). **B** Maximum absorbance (A_460_, a.u.) reached for each concentration of formamide*.* The reaction of formamide was catalyzed by formamidase (AmiF) and formate dehydrogenase (FDH). The reaction proceeded at 37 °C. The reaction mixture (0.2 mL) contained 0.02 mL of Tris/HCl buffer (1 mol/L, pH 7.1), AmiF (1 U), FDH (0.2 U), NAD + (5 mmol/L), WST-8 (0.2 mmol/L), and 1-mPMS (0.008 mmol/L); 0.1 mL of formamide solution (pH 9.5) was added to a final concentration of 0.005–0.10 mmol/L

For the alternative cascade starting with CynH, we first investigated the performance of the second step catalyzed by AmiF and FDH. Formamide was used as a substrate. The absorbance at 460 nm reached its maximum after 20 to 30 min (Fig. 3A). The response was almost linear for the whole range of the formamide concentrations used (0.005 through 0.10 mmol/L, Fig. 3B).

The calibration was constructed by plotting the maximum absorbance achieved for each concentration against the concentration of formamide (Fig. 3B). The LOD and LOQ were calculated to be 13.1 μmol/L (0.59 mg/mL) and 39.8 μmol/L (1.79 mg/mL), respectively. Having confirmed that this cascade is feasible, we combined it with the fCN hydration by a CynH to develop the second fCN assay (see below). This reaction could also serve for the determination of formamide which is used for softening paper, glues and gums, and in chemical synthesis (https://pubchem.ncbi.nlm.nih.gov/compound/Formamide).

### Determination of free cyanide using a cyanide hydratase–formamidase–formate dehydrogenase cascade

**Fig. 4:**
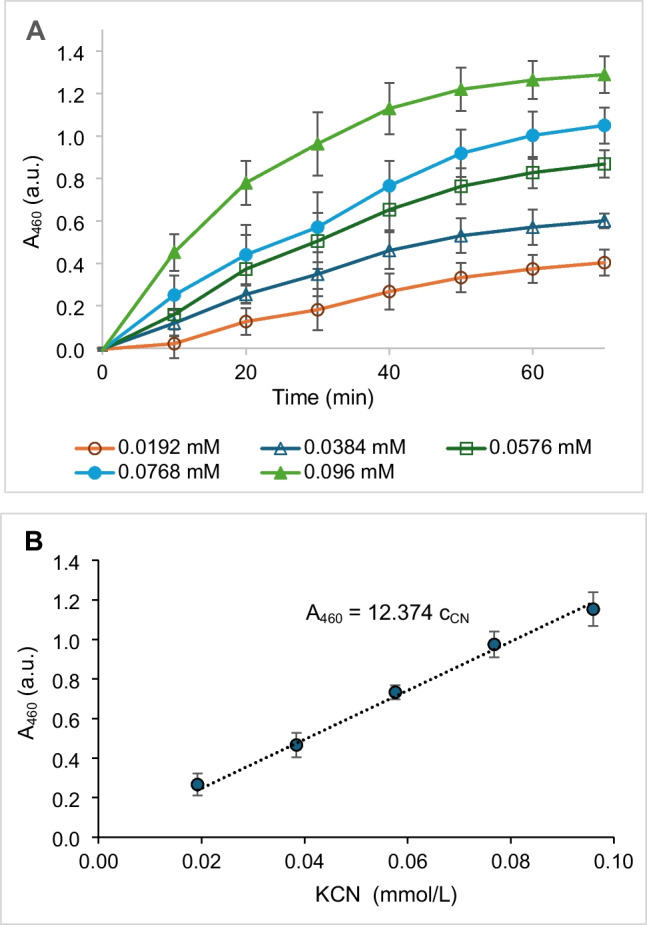
**A** Development of absorbance of formazan at 460 nm (A_460_, absorbance units, a.u.) in the reactions of various concentrations of KCN (c_CN_, mmol/L). **B** Maximum absorbance (A_460_, a.u.) reached for each concentration of KCN. The reaction of KCN was catalyzed by cyanide hydratase (CynH), formamidase (AmiF), and formate dehydrogenase (FDH). The first step proceeded at 30 °C. The reaction mixture (0.5 mL) contained glycine/NaOH buffer (100 mmol/L, pH 9.5), KCN (0.0192–0.096 mmol/L), and CynH (10 U). The second step proceeded at 37 °C. The reaction mixture (0.20 mL) contained 0.12 mL of sample from the first step, 0.02 mL of Tris/HCl buffer (1 mol/L, pH 7.1), AmiF (1 U), FDH (0.2 U), NAD^+^ (5 mmol/L), WST-8 (0.2 mmol/L), and 1-mPMS (0.008 mmol/L)

In this assay, the formamide standards in the assay mixture (see the previous section) were replaced with samples from the hydration of KCN by CynH. Other conditions were the same as in the above assay. The absorbance at 460 nm was read out within 70 min (Fig. 4A). The increase in the absorbance between 60 and 70 min was minor, but it is likely that the absorbance would still slightly increase after prolonged reaction times. The response was almost linear for KCN concentrations between approximately 0.02 and 0.10 mmol/L (Fig. 4B), and it was higher than in the cascade starting with the CynD (see above). We assume this could be due to a higher conversion of substrate that was achieved with the CynH in comparison with CynD. The maximum concentration of NADH produced by CynD + FDH was between 42 and 61% of the theoretical concentration that could be obtained from the KCN standards (see Electronic Supplementary Material, Table S1). This indicated the conversion of fCN or formic acid was incomplete. In contrast, the concentrations of NADH produced by CynH + AmiF + FDH were largely in a good accordance with the theoretical concentration that could be produced from the standards (99–116% of the theoretical NADH concentration, Table S1). These data suggest the complete conversion of the substrate. Although both CynD and CynH are active at pH 9.5, CynD may be less stable under these conditions, and its activity may diminish during the reaction. Therefore, the biochemical properties of CynD need to be further investigated. Anyway, this study indicates that the three-enzyme reaction provides a higher response.

Overall, the LOD and LOQ (Table 1) were the lowest for the cascade starting with the CynH: 7.00 µmol/L (0.18 mg/L) and 21.2 µmol/L (0.55 mg/L), respectively. Thus, in this case, the LOD is below the US Environmental Protection Agency (EPA) limit for cyanide in drinking water, i.e., 0.2 mg/L [30].

When comparing the proposed assays with current cyanide assays, the results should be seen in a broader context, taking into account the sensitivity but also the possible use of harmful chemicals or harsh reaction conditions and interferences. In comparison with the picric acid method, a routine fCN assay detecting ≥ 1 mg fCN/L [5], the enzymatic assay is over five times more sensitive. In addition, the enzymatic approach avoids the use of hazardous chemical and harsh conditions, which are the disadvantages of the picric acid method. In contrast, the Spectroquant kit allows to determine lower levels of cyanide such as 0.01 mg/L in a 1-cm cuvette. However, the kit is not designed for microplate readers and requires large amounts of reagents. Another disadvantage of the kit is its sensitivity to certain interfering ions (e.g., Br^−^, NO_2_^−^, SCN^−^, Ag^+^, Cu^2+^, Hg^2+^, Ni^2+^) [8].

The enzymatic fCN assays based on fCN hydrolysis to formic acid are selective by principle, although formic acid is naturally an interfering compound. However, this disadvantage can be surpassed by performing a control reaction without formamidase or without the cyanide-transforming enzyme. Certain compounds present in the assayed samples could inhibit the enzymes used. These effects have to be investigated for various types of samples. While studying the degradation of fCN by CynHs, we observed that wastewater components such as sulfides or thiocyanates did not significantly inhibit the enzyme [26], which is encouraging. Sulfides and sulfites (both from 1 mg/L) strongly interfere with the picric acid method, and thiocyanates in a concentration of over 0.05 mg/L affect the results obtained with the above kit, along with other ions [8]. Thiocyanates are usual components of coke-plant wastewaters and occur in them in concentrations up to 0.5 g/L [31], while sulfide concentrations up to 3.5 g/L are found in petrochemical wastewaters [32].

## Conclusions

We report a proof of concept for new colorimetric fCN assays based on a sequential enzymatic conversion of fCN connected to NADH formation. Both variants of the enzyme cascade starting either with CynD or CynH were possible. However, a higher response and hence a lower LOD and LOQ were achieved with the latter. The amount of NADH produced is proportional to fCN converted and is determined using a sensitive method employing color formazan as the final product. Formamide, an important industrial chemical, can be determined analogously. The assays are in a high-throughput format, and the consumption of enzymes and chemicals is minimal. The CynD, CynH, or AmiF enzymes, unlike FDH (commercial), were overproduced and purified by us, and their preparation was uncomplicated. The method is appropriate for on-site use, and its sensitivity will probably be sufficient for many applications, such as the analysis of industrial wastewaters or detection of cyanide spills. The suitability of the method for specific applications will be investigated in the future.

## Supplementary Information

Below is the link to the electronic supplementary material.

Supplementary file1 (DOCX 588 KB)
