# Correction to: Design and development of spectrophotometric enzymatic cyanide assays

**DOI:** 10.1007/s00216-025-05853-9

**Published:** 2025-04-01

**Authors:** Katarína Šťastná, Ludmila Martínková, Lenka Rucká, Barbora Křístková, Romana Příhodová, Pavla Bojarová, Miroslav Pátek

**Affiliations:** 1 Institute of Microbiology of the Czech Academy of Sciences, CZ‑142 00 Prague, Czech Republic; 2 Department of Biochemistry, Faculty of Sciences, Charles, University, CZ‑128 44 Prague, Czech Republic; 3 Faculty of Food and Biochemical Technology, University of Chemistry and Technology, Prague, CZ‑166 28 Prague, Czech Republic; 4 Department of Health Care Disciplines and Population Protection, Faculty of Biomedical Engineering, Czech Technical University in Prague, nam. Sitna 3105, CZ‑272 01 Kladno, Czech Republic

**Correction to: Analytical and Bioanalytical Chemistry (2025) 417:697–704**

10.1007/s00216-024-05703-0

The article Design and development of spectrophotometric enzymatic cyanide assays, written by Katarína Šťastná, Ludmila Martínková, Lenka Rucká, Barbora Křístková, Romana Příhodová, Pavla Bojarová, and Miroslav Pátek, was originally published Online First without Open Access. After publication in volume 417, issue 4, pages 697–704 the author decided to opt for Open Choice and to make the article an Open Access publication. Therefore, the copyright of the article has been changed to © The Author(s) 2025 and the article is forthwith distributed under the terms of the Creative Commons Attribution 4.0 International License, which permits use, sharing, adaptation, distribution and reproduction in any medium or format, as long as you give appropriate credit to the original author(s) and the source, provide a link to the Creative Commons licence, and indicate if changes were made. The images or other third party material in this article are included in the article’s Creative Commons licence, unless indicated otherwise in a credit line to the material. If material is not included in the article’s Creative Commons licence and your intended use is not permitted by statutory regulation or exceeds the permitted use, you will need to obtain permission directly from the copyright holder. To view a copy of this licence, visit http://creativecommons.org/licenses/by/4.0/.

The original article has been corrected.

