# Supplementary material for: Design and development of spectrophotometric enzymatic cyanide assays
Source: Anal Bioanal Chem. 2024 Dec 20;417(4):697–704. doi: 10.1007/s00216-024-05703-0 (PMC11954721; doi:10.1007/s00216-024-05703-0)
Supplement: Supplementary file 1 — Supplementary file1 (DOCX 588 KB) [file 216_2024_5703_MOESM1_ESM.docx]

**Electronic Supplementary Material**

# Design and development of spectrophotometric enzymatic cyanide assays

**Katarína Šťastná ^a,b^, Ludmila Martínková ^a*^, Lenka Rucká ^a^, Barbora Křístková ^a,c^, Romana Příhodová ^a^, Pavla Bojarová ^a,d^, Miroslav Pátek ^a^**

^a^ Institute of Microbiology of the Czech Academy of Sciences, CZ-142 00 Prague, Czech Republic

^b^ Department of Biochemistry, Faculty of Sciences, Charles University, CZ-128 44 Prague, Czech Republic

^c^ Faculty of Food and Biochemical Technology, University of Chemistry and Technology, Prague, CZ-166 28 Prague, Czech Republic

^d^ Department of Health Care Disciplines and Population Protection, Faculty of Biomedical Engineering, Czech Technical University in Prague, nám. Sítná 3105, CZ-272 01 Kladno, Czech Republic

*Content:*

**Table S1** Concentration of NADH produced by the enzymatic conversion of free cyanide

**Fig. S1** SDS-PAGE analysis of cyanide dihydratase CynD_pum-stut_

**Fig. S2** Spectrophotometric determination of NADH

**Table S1** Concentration of NADH produced by the conversion of free cyanide

| **Initial concentration of fCN (mmol/L)** | **NADH (mmol/L) / % of theoretical concentration produced** | |
| --- | --- | --- |
|  | **by CynD+FDH** | **by CynH+AmiF+FDH** |
| 0.019 | 0.008 / 42 | 0.022 / 116 |
| 0.038 | 0.020 / 53 | 0.038 / 100 |
| 0.058 | 0.030 / 52 | 0.060 / 103 |
| 0.077 | 0.047 / 61 | 0.080 / 104 |
| 0.096 | 0.052 / 54 | 0.095 / 99 |

AmiF, formamidase; fCN, free cyanide; FDH, formate dehydrogenase; CynD, cyanide dihydratase; CynH, cyanide hydratase

**
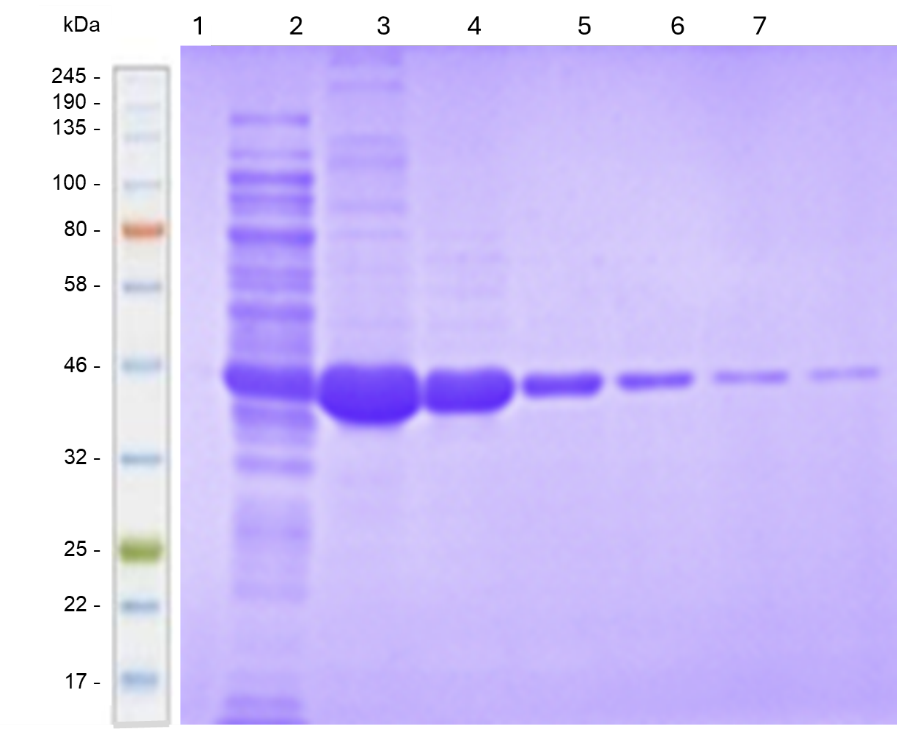
**

**Fig. S1.** SDS-PAGE analysis of cyanide dihydratase CynD_pum-stut_. The purification was monitored by SDS-polyacrylamide gel: lane 1, cell-free extract; lane 2, fraction eluted with 50 mmol imidazole/L; lane 3-6, fractions eluted with 200 mmol imidazole/L; lane 7, fraction eluted with 400 mmol imidazole/L.

| **A** |  |  |
| --- | --- | --- |
| **B** |  |  |

**Figure S2.** (**A**) Determination of NADH at 340 nm: calibration curve for 0-0.5 mmol NADH/L. (**B**) Determination of NADH at 460 nm: calibration curve for 0-0.1 mmol NADH/L. All measurements were carried out using a microtitration plate reader Tecan Sunrise^TM^ controlled by the software Magellan^TM^ (Tecan).
